# mGlu2 Receptor Agonism, but Not Positive Allosteric Modulation, Elicits Rapid Tolerance towards Their Primary Efficacy on Sleep Measures in Rats

**DOI:** 10.1371/journal.pone.0144017

**Published:** 2015-12-11

**Authors:** Abdallah Ahnaou, Hilde Lavreysen, Gary Tresadern, Jose M. Cid, Wilhelmus H. Drinkenburg

**Affiliations:** 1 Dept. of Neuroscience, Janssen Research & Development, A Division of Janssen Pharmaceutica N.V., Turnhoutseweg 30, B-2340, Beerse, Belgium; 2 Neuroscience Medicinal Chemistry, Janssen Research & Development, Janssen-Cilag S.A., Jarama 75, Polígono Industrial, 45007, Toledo, Spain; University of North Dakota, UNITED STATES

## Abstract

G-protein-coupled receptor (GPCR) agonists are known to induce both cellular adaptations resulting in tolerance to therapeutic effects and withdrawal symptoms upon treatment discontinuation. Glutamate neurotransmission is an integral part of sleep-wake mechanisms, which processes have translational relevance for central activity and target engagement. Here, we investigated the efficacy and tolerance potential of the metabotropic glutamate receptors (mGluR2/3) agonist LY354740 versus mGluR2 positive allosteric modulator (PAM) JNJ-42153605 on sleep-wake organisation in rats. In vitro, the selectivity and potency of JNJ-42153605 were characterized. In vivo, effects on sleep measures were investigated in rats after once daily oral repeated treatment for 7 days, withdrawal and consecutive re-administration of LY354740 (1–10 mg/kg) and JNJ-42153605 (3–30 mg/kg). JNJ-42153605 showed high affinity, potency and selectivity at mGluR2. Binding site analyses and knowledge-based docking confirmed the specificity of JNJ-42153605 at the mGluR2 allosteric binding site. Acute LY354740 and JNJ-42153605 dose-dependently decreased rapid eye movement (REM) sleep time and prolonged its onset latency. Sub chronic effects of LY354740 on REM sleep measures disappeared from day 3 onwards, whereas those of JNJ-42153605 were maintained after repeated exposure. LY354740 attenuated REM sleep homeostatic recovery, while this was preserved after JNJ-42153605 administration. JNJ-42153605 enhanced sleep continuity and efficiency, suggesting its potential as an add-on medication for impaired sleep quality during early stages of treatment. Abrupt cessation of JNJ-42153605 did not induce withdrawal phenomena and sleep disturbances, while the initial drug effect was fully reinstated after re-administration. Collectively, long-term treatment with JNJ-42153605 did not induce tolerance phenomena to its primary functional effects on sleep measures, nor adverse effects at withdrawal, while it promoted homeostatic recovery sleep. From the translational perspective, the present rodent findings suggest that mGluR2 positive allosteric modulation has therapeutic potential based on its superior long term efficacy over agonists in psychiatric disorders, particularly of those commonly occurring with REM sleep overdrive.

## Introduction

Abnormalities in glutamate balance have been recently implicated in the mechanisms underlying neuropsychiatric illnesses. The glutamate signalling through the metabotropic glutamate receptor 2 (mGluR2) is actively pursued in academia and pharmaceutical groups as a promising therapeutic approach to normalize excessive glutamate flow [1–9]. Given the potential for induction of tolerance with GPCR agonists, an important question remains unanswered regarding efficacy and safety following longer term use of the mGluR2 agonist, as well as the duration of its effectiveness. Preclinical studies have reported behavioral data with differential effects regarding the potential for tolerance development following chronic dosing with mGluR2 agonists, depending on behavioural pharmacology assessed: LY379268 had motor depressant effect, to which animals developed rapid tolerance following repeated administration [10–11]. In drug abuse model, repeated administration of LY379268 reduced toluene-induced hyperlocomotion [12], whereas repeated dosing with LY379268 had no effect on PCP-induced hyperlocomotor activity [10]. In addition, subchronic treatment with LY354740 failed to reverse ketamine-evoked prepulse inhibition deficits and hyperlocomotion [6]. Moreover, acute LY379268 was effective in pain models; while tolerance developed against its analgesic effect upon repeated dosing [13].

In mammals and submammalian species, sleep is regulated by homeostatic and circadian factors. The brain structures actively involved in waking are located in the basal forebrain, hypothalamus and brainstem pons, whereas the active sleep mechanisms mainly reside in the preoptic/anterior hypothalamic area. Glutamate is, in addition to acetylcholine, monoamines and hypocretin/orexin, an important factor with dual function in waking and sleeping. Glutamate release follows daily fluctuation rhythms, with peak levels during waking and Rapid Eye Movement (REM) sleep. Suprachiasmatic nucleus (SCN) projections to the ventromedial and ventrolateral regions of the preoptic area use both glutamate and GABA as stimulatory and inhibitory inputs, respectively for the control of the sleep-wake rhythms [14]. The glutamate input from SCN to paraventricular neurons [15,16] is believed to stimulate melatonin synthesis [17]. In addition, microdialysis studies demonstrated an enhancement of glutamate release in the orbitofrontal cortex during REM sleep in rats [18]. Moreover, real-time biosensor measurement of neurotransmitters release across the sleep-wake cycle revealed increases in glutamate level in the pre-frontal cortex during wakefulness, decreases during sleep episodes and spike increases during REM sleep [19]. Furthermore, several research groups investigated the effects of mGluR modulation on sleep [20–24]. mGluR2’s are highly expressed in the limbic amygdaloid nuclei [25–27], known to play a pivotal role in the regulation of REM sleep [23,28]. The role of mGluR2 in the regulation of the sleep-wake cycle has been supported by using specific pharmacological agents on this receptor. Great consistency was found across all studies with respect to the suppressing effect on REM sleep following the activation of mGluR2 [20,21,23], whereas blockade of mGluR2 elicited waking [21,29]. The specific suppression effect on REM sleep has been confirmed in WT but not mGluR2 (-/-) mice [20]. Collectively, these studies provide evidence for a strong relationship between glutamate neurotransmission and sleep mechanisms. In addition, empirical data showed a clear-cut relationship between sleep disturbances and mental disorders involving glutamatergic abnormalities, which lends credence for the study of glutamate abnormalities as a potential common factor. Therefore, sensitive experimental paradigms such as the sleep-wake model can be advantageous applied in translational research to screen potential mGluR2-based therapeutic compounds.

Recently we have developed a specific PAM JNJ-42153605 with good metabolic stability, displaying high in vitro potency and good selectivity for the mGluR2 with an acceptable pharmacokinetic profile in both rodent and non-rodent species [30]. The present studies were aimed at investigating whether chronically activated mGluR2 results in rapid tolerance towards its primary functional effects on sleep and arousal behavior. To this end, after characterisation of the test compound’s specificity, selectivity, molecular binding and docking characteristics, the efficacy and tolerance potential of effects on sleep-wake behaviour in rats after once daily dosing was compared between the mGluR2/3 agonist LY354740 and the PAM JNJ-42153605.

## Material and Methods

### 1. In vitro pharmacology

For functional [^35^S]GTPγS binding, Chinese Hamster Ovary (CHO) cells expressing the human or rat mGluR2 were grown until 80% confluence, washed in ice-cold phosphate-buffered saline and homogenized. Protein concentrations were measured by the Bio-Rad protein assay using bovine serum albumin as standard. Final assay mixtures contained 7 (human mGluR2) or 10 (rat mGluR2) μg of membrane protein were pre-incubated with JNJ-42153605 alone (determination of agonist effects) or together with an EC_20_ concentration (4 μM) of glutamate (determination of PAM effects) for 30 min at 30°C. [^35^S]GTPγS was added at a concentration of 0.1 nM and Filter-bound radioactivity according to an earlier protocol [31].

For mGluR2 competition binding, membranes from human mGluR2-CHO cells were homogenized and suspended in assay mixtures with 2 nM [^3^H]LY341495 and appropriate concentrations of JNJ-42153605. Non-specific binding was determined in the presence of 1 mM glutamate and was about 10% of total binding.

For mGluR2 selectivity, Ca^2+^ assays (Fluorescent Drug Screening system, FDSS, Hamamatsu) were used to assess the activity at the human mGluR1, 3, 5, 7 or 8 receptor and [^35^S]GTPγS binding experiments were performed to evaluate the activity of JNJ-42153605 on the rat mGluR6 and human mGluR4 using the protocol described elsewhere [31].

JNJ-42153605 was furthermore tested for its inhibition of radioligands binding to a battery of neurotransmitter and peptide receptors, ion channels and transporters (CEREP, Celle L’Evescault, France).

### 2. Building an mGluR2 receptor homology model

A model of the active state 7TM domain of the human mGluR2 (Uniprot code Q14416) bound to G protein was built using several structural templates. The crystal structure of human mGluR5 (PDB 4OO9, [32]) was used to model all 7TM helices except TM6. The β_2_AR (PDB ID 3SN6, [33]) active structure was used to model both the distinct open TM6 conformation as well as the G protein. Extracellular loop 2 (ECL2) is not refined in the mGluR5 X-ray structure and was therefore modelled based on the mGluR1 crystal structure (PDB 4OR2, [34]. The sequence alignment for the model building is provided in S1 Fig. The mGluR2 7TM monomer can be activated upon PAM binding [35]. Overall sequence identity between mGluR2 and mGluR5 7TM’s was 51%. The initial model was constructed in MOE v2014.9 (Chemical computing group Inc., Montreal, QC, Canada) and then Maestro (Schrodinger LLC, New York, NY, USA) was used for structure preparation. Amino acid numbering is based on recent recommendations [36].

#### 2.1 Docking of JNJ-42153605

The ligand was prepared for docking using Maestro. Conformational sampling was performed with ConfGen and multiple conformers were docked into the mGlu_2_ active state model using Glide XP. The docking grid was centered on the ligand position in the mGluR1 structure. Sampling was increased in the Glide docking by turning on expanded sampling and passing 100 initial poses to post-docking minimisation. All other docking parameters were set to the defaults.

### 3. In vivo pharmacology

#### 3.1 Animals, surgery and polysomnography recordings

All animal studies have been carried out in accordance with guidelines of the Association for Assessment and Accreditation of Laboratory Animal Care International (AAALAC), and of the European Communities Council Directive of 24 November 1986 (86/609/EEC) and were approved by Janssen Pharmaceutica Ethical Committee. Every effort was made to minimize animal use and disturbances in animal well-being and experimental animals were euthanized at the end of the study by common rodents CO2 procedure. Sixty-four male Sprague Dawley rats (Crl:SD, Charles River, France) were housed in controlled environmental conditions throughout the study: 22°C ± 2°C ambient temperature, relative humidity 60%, standard 12:12 light cycle regime (lights on from 12:00 a.m. to 12:00 p.m., illumination intensity: ~ 100 lux (i.e. at the height of the recording box, while a recessed lighting consoles managed diffuse and uniform light levels below 60 lux within cages, a software-controlled dimmer handled a gradual transition between light and dark cycles). Standard rodent pellets and tap water were provided *ad libitum*.

Surgery was performed using the protocol described earlier [20]. In short, a mixture of 30% O2, 70% N2O and 5% isoflurane was administered to animals as an initial induction for 2 minutes. Then, the animals were mounted in a stereotaxic apparatus and were given a continuous constant mixture of O2, N2O and 2% isoflurane. An analgesic Piritramide (dipidolor) was administered before the incision over the total length of the head. The oval area of the scalp was removed, and the uncovered skull was cleared of the periosteum in order to place 4 fixing stainless steel screws for the recording of the frontal and parietal electroencephalogram (EEG). For the recording of the electro-oculogram (EOG) and electromyogram (EMG), stainless steel wires were placed in peri-orbital and into the nuchal muscle, respectively. Electrodes were fitted into an 8 holes connector and were fixed with dental cement to the cranium.

After a recovery period of at least 10 days, animals were gently handled twice a day for one week: rats were quietly held and gently stroked by hand and were increasingly allowed to explore and become familiar with the experimenter’s hands. Afterwards, the rats were systematically habituated to their surroundings and recording procedure. These habituation procedures enable a reduction in anxiety and stress that may occur during the experimental procedure and therefore reduce subsequent consequences for data collection. A welfare monitoring of each animal was managed by using an animal inventory system taking to consideration regular general observations of behavior and physical health. During the adaptation period and recordings, animals were carefully connected via a rotating swivel to a bipolar recorder amplifier (Embla, MedCare Flaga, Iceland) for recording of polygraphic signals with an input range of +/- 500 mV.

All experiments were performed in a large scale EEG laboratory setting under controlled conditions, in which animals were kept in their home cage placed in recording boxes six hours before the start of the first baseline recording and throughout the chronic experiments in order to avoid any stress that may result from cage changes and displacement of home-cages from the holding room to recording room and vice-versa. The first recording session started at 14:00 pm (i.e. end of the second hour of the light period) and lasted 20-h after the administration of saline in vehicle and drug-treated groups. The selection of this post-acrophase of sleep for the timing of pharmacological administration, is taken as phase reference when sleep pressure is neither maximal nor minimal, thus allowing for assessment and observation of subtle drug effects on sleep parameters. The consecutive recordings were performed for the same duration and circadian conditions following repeated treatments. In the first group of animals (n = 32), polygraphic recordings were performed during 7 consecutive days following once daily administration of LY354740 (1, 3 and 10 mg/kg) and of vehicle for the control group (n = 8 for each condition). In the second group of animals (n = 32), recordings were performed during 7 consecutive days following once daily administration of JNJ42153605 (3, 10 and 30 mg/kg) and of vehicle for the control group (n = 8 for each condition), during the 3 days after withdrawal, and during 2 subsequent days after re-administration of the compound or vehicle.

Although the level of occupancy required for REM inhibition may be different for each pharmacological mechanism, the dose-ranges for both compounds were selected to ensure equipotent dose effects for JNJ-42153605 and LY354740 on REM sleep inhibition. Given the relative short half-life (T_1/2_) values of both drugs given at a single dose (JNJ42153605 at 10 mg/kg: 2.7 ± 0.2-h; and LY354740 at 1 mg/kg: 0.91 ± 0.3-h), a once 24-h dosing schedule allows for comparing development of tolerance while taking pharmacokinetic parameters into consideration.

#### 3.2 Vigilance states analysis

A sleep-wake analysis system was applied for 20 continuous hours following acute and sub-chronic administration of LY354740 or JNJ-42153605. As previously described [20], the discriminative analysis uses classification rules to assign the sleep-wake stages based on 6 EEG frequency domain values (δ: 0.5–4 Hz, θ: 4.2–8 Hz, α: 8.2–12 Hz, σ: 12.2–14 Hz, β: 14.2–30 Hz, γ: 30.2–50 Hz), integrated EMG, EOG and body activity level.

Six vigilance states were classified as being indicative of respectively active wakefulness, passive wakefulness, light sleep, deep sleep, intermediate stage or REM sleep. Briefly, different vigilance states were characterized as follows: Active wake: low-voltage fast EEG activity, high EMG activity, numerous eye movements and high body activity; Passive wake: low-voltage fast EEG activity, high to moderate EMG activity, numerous eye movements and absence of body activity; Light sleep: high-voltage slow cortical waves interrupted by low-voltage fast waves and reduced EMG activity; Deep sleep: continuous high-amplitude slow-wave activity in EEG in absence of EMG, EOG and body activity; Intermediate sleep: transient spindle activity with theta rhythm, absence of EOG and body movements; REM sleep: low-voltage fast cortical waves with a regular theta rhythm, presence of rapid eye movements and absence of muscular and body movements. The scores were synchronized in time with the EEG signal and different sleep-wake parameters were calculated, such as the amount of time spent in each vigilance state, the number and duration of episodes in each state, latencies for deep sleep and REM sleep and the number of shifts from one state to another one. For each sleep state, the latency was defined as the time between the beginning of the recording and the appearance of the first sleep period lasting at least 20 consecutive seconds. To determine whether LY354740- and JNJ-42153605 induced inhibition of REM sleep during the light phase would influence the pattern of late REM sleep recovery, the total time spent in REM sleep over the entire dark phase of the circadian time were assessed.

### 4. Drugs

(+)-2-aminobicyclo [3.1.0]hexane-2,6-dicarboxylate (LY354740) and 1,2,3-triazolo[4,3-a]pyridine (JNJ-42153605) were synthesized at Janssen Research and Development laboratories. For oral administration in vivo studies, LY354740 was prepared in 10% Cyclodextrin + NaoH, while JNJ-42153605 was dissolved in 20% Cyclodextrin + 1HCl. All drugs were given at a volume of 10 ml/kg of body weight in rat. An equivalent volume of vehicle was administered in control animals.

### 5. Statistical analysis

The time course of different sleep variables following drug treatment were expressed as the mean ± S.E.M averaged within each treatment group and presented as mean values over periods of 60-min. The Wilcoxon Mann–Whitney Signed Rank test with Bonferroni correction tests was used to compare the values of sleep parameters. Probabilities of less than 0.05 were considered statistically significant. Some derived sleep variables (e.g. REM sleep time) were calculated over 4-h post-treatment: the rational for this 4-h period is based on pharmacokinetic parameters such as half-lives of both compounds indicating that direct effects were expected to occur over this time interval. In addition, a mixed-model ANOVA was used to further analyse the dose-response changes in time spent in each vigilance states. Post hoc tests were done comparing the treatment group (drug) to the reference treatment level (vehicle) at each time point. Raw p-values were computed and adjusted for multiplicity by false discovery rate procedure.

## Results

### 1. In vitro pharmacology

**Fig 1:**
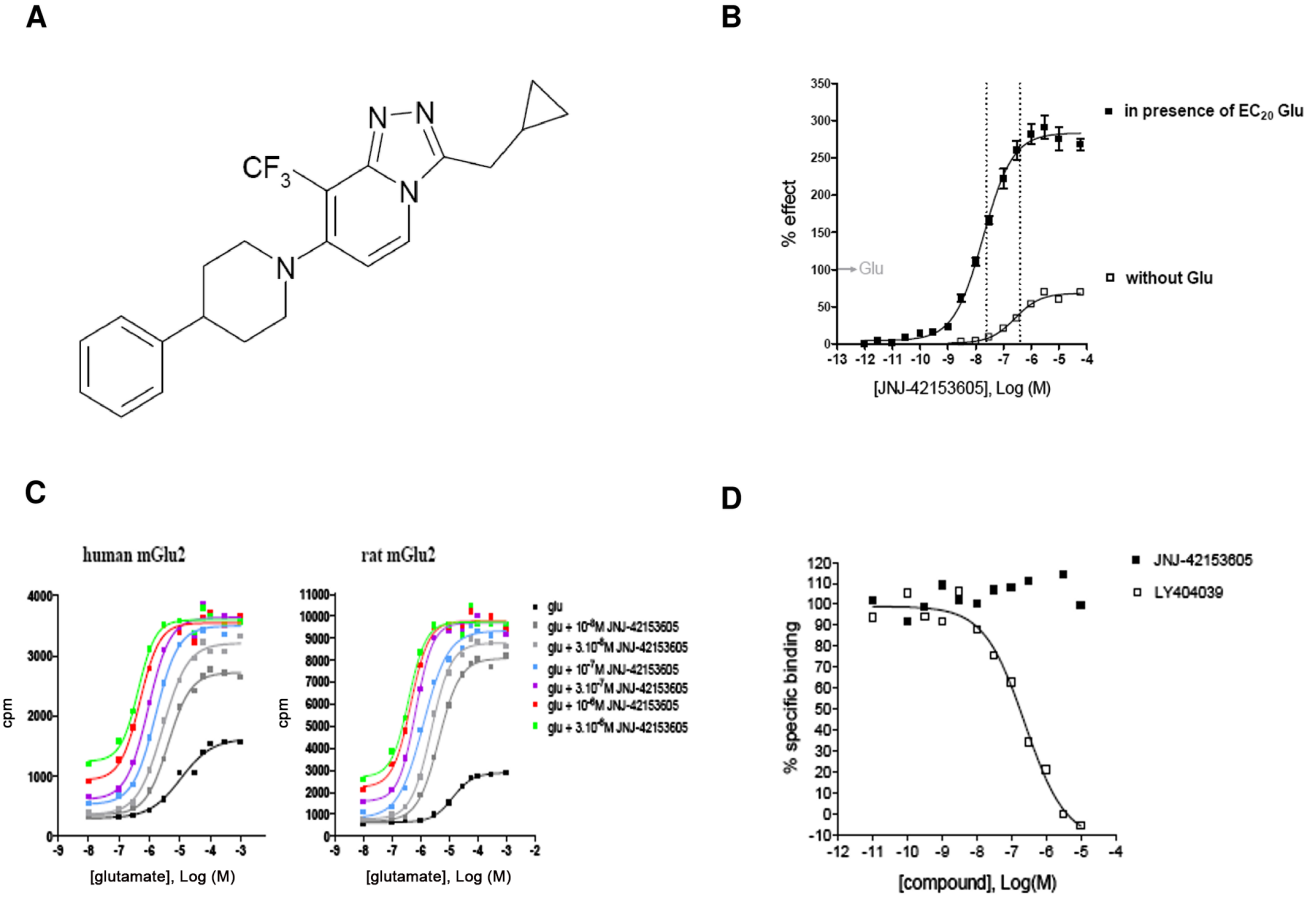
Chemical structure of the mGluR2 PAM JNJ42153605 (A) and in vitro pharmacological characterization thereof *in vitro* in CHO cells expressing the human and/or rat mGluR2 (B, C, D). Fig 1B shows the [^35^S]GTPγS concentration-response curve of JNJ-42153605 in the absence or presence of an EC_20_ glutamate concentration. Data are expressed as percentage of the maximal response to glutamate and are mean ± S.E.M. of 9–10 experiments. Fig 1C represents glutamate-induced [^35^S]GTPγS binding in the absence or presence of increasing concentrations of JNJ-42153605. JNJ-42153605 shifts the glutamate concentration-response curve to the left in CHO cells expressing the human (left panel) or rat (right panel) mGluR2. Data for the human mGluR2 were repeated 3 times with similar results. Fig 1D shows [^3^H]LY341495 binding to the human mGluR2 in the presence of either LY404039 or JNJ-42153605. LY404039 but not JNJ-42153605 displaces [^3^H]LY341495 from the orthosteric agonist (glutamate) binding site.

Fig 1A represents the chemical structure of the mGluR2 PAM JNJ-42153605. The pharmacological specificity of JNJ-42153605 was confirmed in binding and functional studies. At the human mGlu2R, JNJ-42153605 potentiates [^35^S]GTPγS binding induced by 4 μM of glutamate (∼EC_20_) up to 285 ± 34%, with an EC_50_ of 17 ± 6 nM (n = 10; Fig 1B). JNJ-42153605 also activates the receptor on its own, although with a lower potency and to a lower extent compared to its treatment combined with low concentrations of glutamate (EC_50_ = 270 ± 65 nM, E_max_ = 67 ± 8%; n = 9).

Consistent with the effects of an allosteric potentiator, JNJ-42153605 shifts the concentration-response curve of glutamate to the left (Fig 1C left panel), increasing the potency of glutamate up to ∼25-fold. At the human mGluR2, the EC_50_ of glutamate decreases from ∼10 to 0.5 μM with the addition of 3 μM JNJ-42153605. Similar results were obtained in [^35^S]GTPγS assays using CHO cells expressing the rat mGluR2 receptor variant (Fig 1C, right panel).

To confirm whether JNJ-42153605 binds at a site distinct from the glutamate recognition site, we evaluated the potency of the compound to displace binding of [^3^H]LY341495, an orthosteric mGluR antagonist [37]. [^3^H]LY341495 binding to the human mGluR2 was inhibited by LY404039, an mGluR2/3 orthosteric agonist, but not by JNJ-42153605 (Fig 1D), clearly indicating that JNJ-42153605 does not bind to the orthosteric mGluR2 binding site.

JNJ-42153605 is a selective mGluR2 PAM, devoid of agonist or antagonist activity at the human mGluR1, 4, 5, 6, 7 or 8 up to 30 mM (data on file). However, JNJ-42153605 showed some hmGluR3 PAM activity (EC_50_ 770 nM), but with an about 50-fold lower potency compared to the hmGluR2. Moreover, JNJ-42153605 was found inactive in a battery of GPCRs and ion channels tested in a broad CEREP profiling (data on file). These results demonstrate that JNJ-42153605 is a highly selective and potent PAM at the mGluR2.

### 2. Docking of JNJ-42153605 into the mGluR2 7TM Model

**Fig 2:**
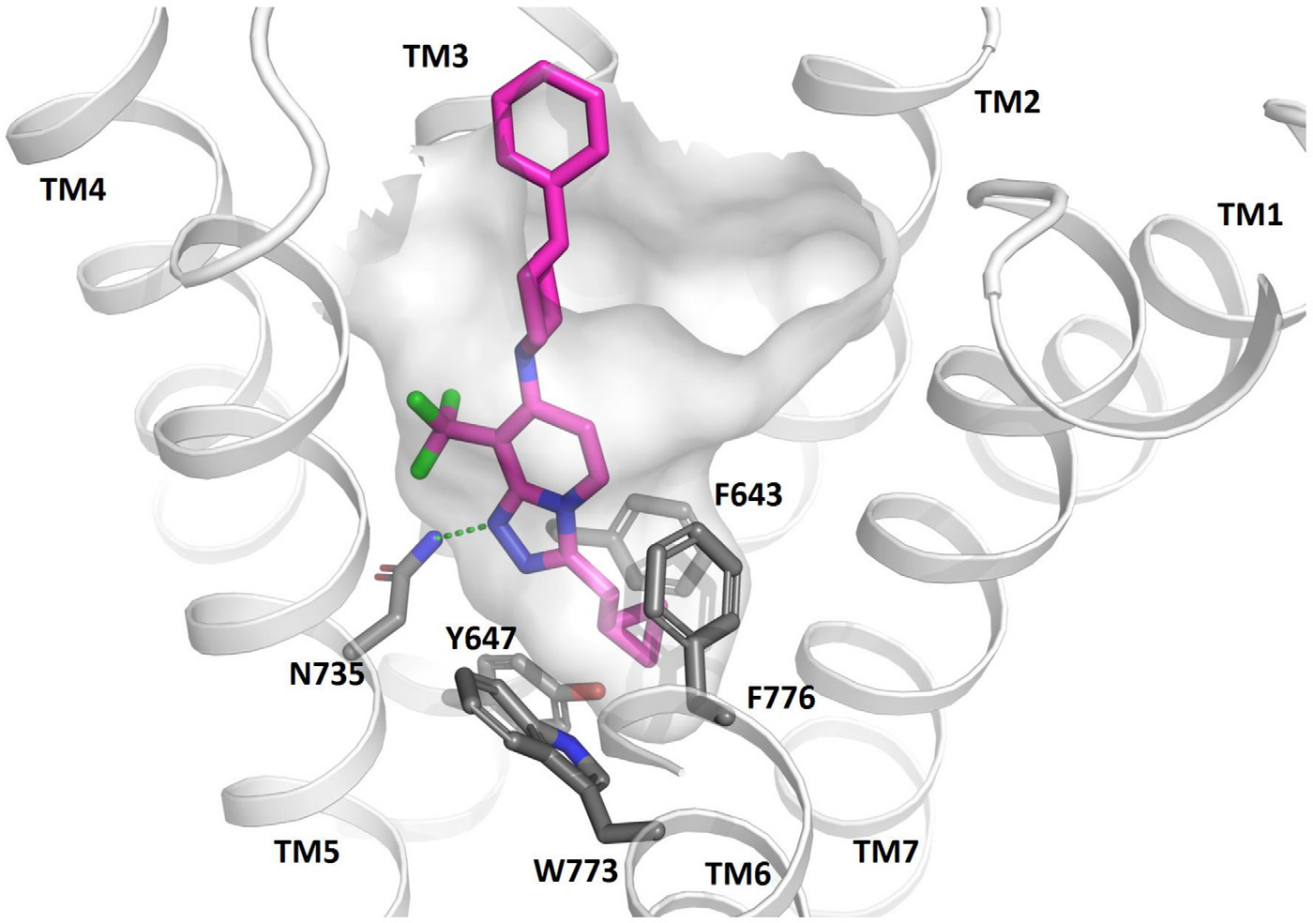
Close up view of the mGluR2 7TM binding site showing the proposed binding mode of JNJ-42153605.

JNJ-42153605 was docked into the active state mGluR2 homology model (Fig 2) and the preferred binding pose is shown. The triazopyridine scaffold binds in the hydrophobic cluster formed between amino acids L639^3.32a.36c^, F643^3.36a.40c^, L732^5.43a.44c^, W773^6.48a.50c^ and F776^6.51a.53c^. The cyclopropylmethyl substituent interacts deeper in the receptor with F776^6.51a.53c^. Aromatic residues L639^3.32a.36c^ and F643^3.36a.40c^ interact on one face of the triazopyridine scaffold and L732^5.43a.44c^ and W773^6.48a.50c^ on the other. Amino acid N735^5.47a.47c^ acts as an H-bond donor to the nitrogen acceptor in the triazo ring of the scaffold. Tryptophan W773^6.48a.50c^ is conserved in the mGluRs and its sidechain adopts an outwards orientation pointing to the membrane in the mGluR5 crystal structure. Further modelling work reveals this sidechain to be flexible and it is shown in Fig 2 in an inwards orientation. The cyclopropyl group makes a steric interaction with W773^6.48a.50c^ at approximately 3 Å distance. The CF_3_ group sits in a small cavity between TM3 and TM5 formed above N735^5.47a.47c^, it interacts with S731^5.42a.43c^. The 4-phenylpiperidine substituent points towards the extracellular side of receptor and the distal phenyl interacts with H723^ECL2^. The predicted binding mode for JNJ-42153605 overlaps with the allosteric site in mGluR1 whereas the mGluR5 modulator goes deeper into the receptor, see S2 Fig.

### 3. In vivo pharmacology

#### LY354740: acute and subchronic effects on sleep-wake measures

**Fig 3:**
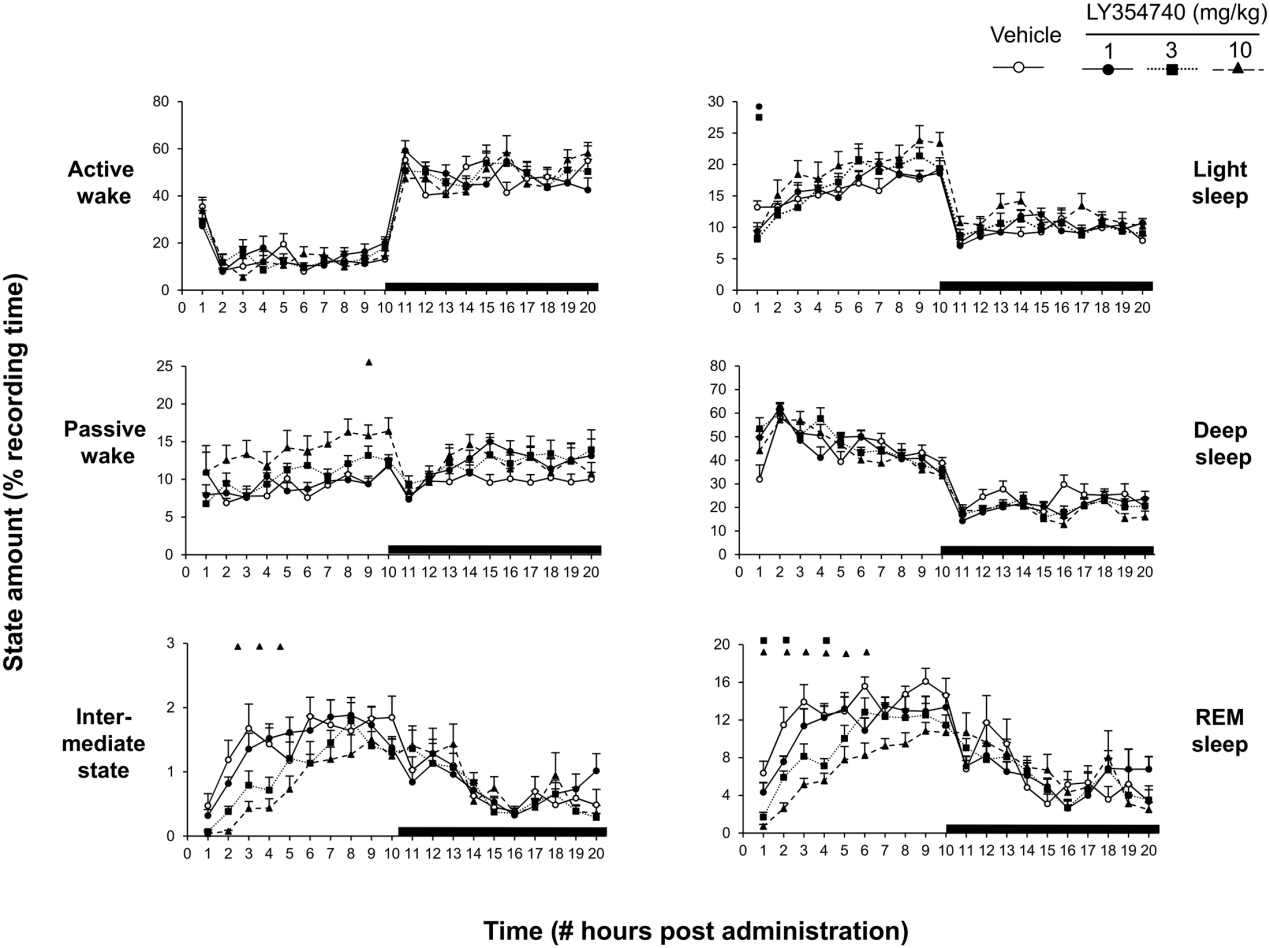
Percentage distribution of the vigilance states active wake, passive wake, intermediate stage, light sleep, deep sleep and rapid eye movement (REM) sleep for each hour period of the 20-h recording session following acute LY354740 (1, 3, 10 mg/kg) or vehicle. Open and dark areas in the abscissa axis indicate light and dark phase of the circadian time, respectively. Values are presented as means ± S.E.M. for each condition expressed in percentage of the recording time. Each symbol indicates statistically significant difference (P < 0.05) between vehicle and drug-dose injected groups.

Treatment day 1: Acute subcutaneous administration of LY354740 (1, 3 and 10 mg/kg) produced significant changes in the distribution of sleep-wake states over the entire recording period, notably a significant decrease in total amount of REM sleep (“treatment x time” interaction: F(12, 1232) = 5.9, p < 0.0001) (Fig 3).

**Fig 4:**
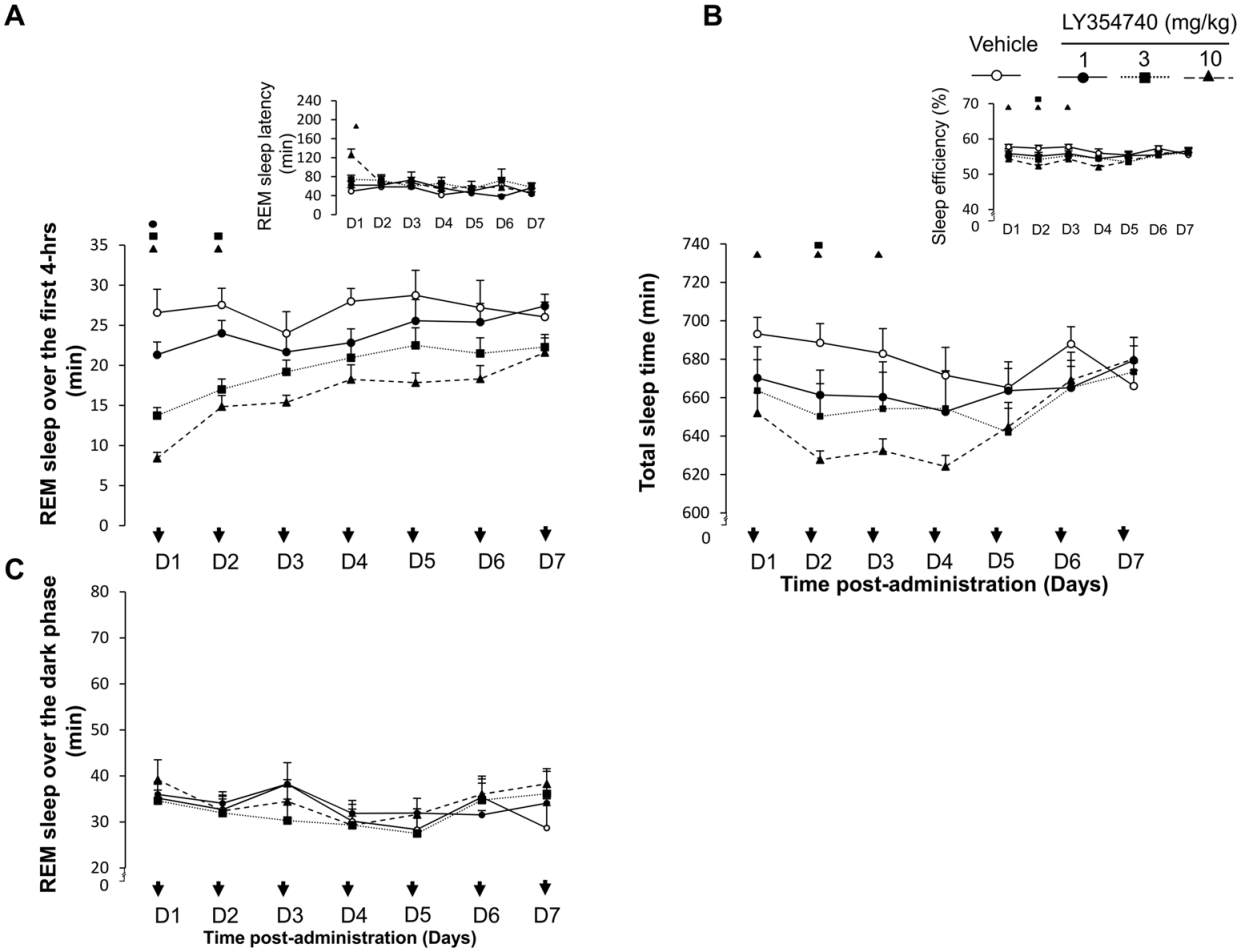
Sleep parameters following repeated dosing with LY354740 (1, 3, 10 mg/kg) or vehicle on experimental days (D1 first treatment day till D7 seventh treatment day). Total sleep time expressed in minutes (Panel A) and sleep efficiency expressed in percentage (Panel A inset). REM sleep amount during the first 4-h of recording sessions after repeated administration (Panel B) and REM sleep latency (Panel B inset). Total amount of REM sleep expressed in minutes that was recovered during the subsequent 10-h dark period (Panel C). The data are presented as means ± S.E.M. Significant differences between the vehicle and LY354740 group are indicated by the drug-dose symbol.

Closer analysis of time distribution among arousal and sleep stages indicated that LY354740 (1, 3, and 10 mg/kg) dose-dependently reduced REM sleep (“treatment x time” interaction: F(9, 212) = 5.9, p < 0.0001) for up to 4-h following dosing on day 1 (Fig 4A).

The suppression of REM sleep was derived from a reduction in both numbers and mean durations of this vigilance state. Consequently, decreases in total sleep time and sleep efficiency resulted from direct effect of LY354740 on REM sleep mechanisms (Fig 4B, inset). Concomitant to changes in REM sleep state, LY354740 consistently lengthened REM sleep onset latency F(3, 28) = 12.8, p < 0.0001) (Fig 4A, inset).

Treatment days 2 till 7: LY354740 consistently reduced the duration of REM sleep up to 4-h post-administration on subsequent day 2 (“treatment” F(3, 28) = 13.3, p < 0.0001), “time” F(3, 212) = 39.4, p < 0.0001), however the ANOVA interaction “treatment x time” did not reach significance level; F(9, 212) = 1.23, p = 0.27) (Fig 4A). Similarly, the intermediate state was reduced (“treatment” F(3, 28) = 6.1, p = 0.0026), time” F(3, 212) = 22.2, p < 0.0001), however, the ANOVA interaction “treatment x time” did not reach significance level; F(9, 212) = 1.24, p = 0 .27) (Fig 4A).

Total sleep time and sleep efficiency were decreased only at the highest dose of the compound. However, as from day 3 onwards LY354740 did not lead to significant changes in total amount of different vigilance states and sleep parameters including REM sleep (Fig 4A). Hence, no withdrawal and re-administration of the mGluR2 was considered afterwards.

During the 10-h recovery dark period, LY354740’s treated rats had similar total amount of REM sleep recovered to that shown by the vehicle treated animals.

#### JNJ-42153605: acute and subchronic effects on sleep-wake measures

**Fig 5:**
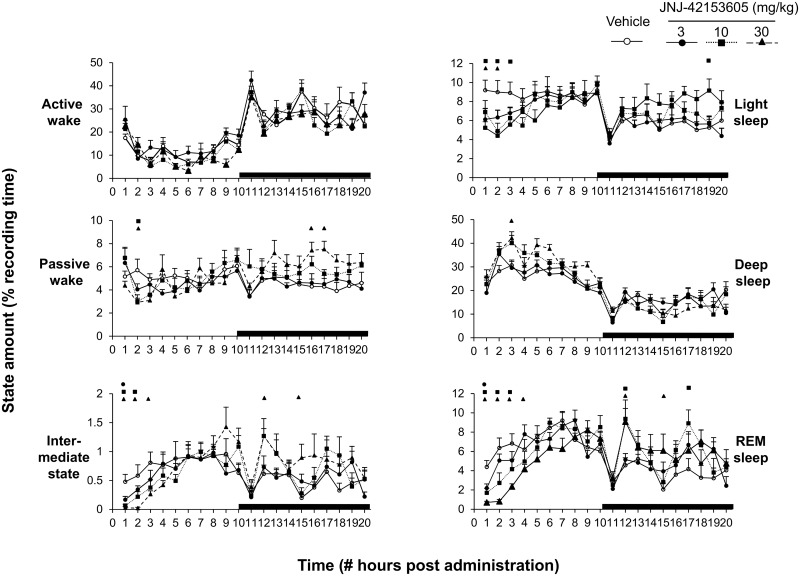
Percentage distribution of the vigilance states active wake, passive wake, intermediate stage, light sleep, deep sleep and rapid eye movement (REM) sleep for each hour period of the 20-h recording session following acute JNJ-42153605 (3, 10, 30 mg/kg) or vehicle. Open and dark areas in the abscissa axis indicate light and dark phase of the circadian time, respectively. Values are presented as means ± S.E.M. for each condition expressed in percentage of the recording time. Each symbol indicates statistically significant difference (P < 0.05) between vehicle and drug-dose injected groups.

Treatment day 1: Oral administration of JNJ-42153605 (3, 10 and 30 mg/kg) significantly reduced REM sleep (“treatment x time” interaction: F(12, 1232) = 10.6, p < 0.0001) and intermediate stage sleep (“treatment x time” interaction: F(12, 1232) = 14.3, p < 0.0001) (Fig 5).

**Fig 6:**
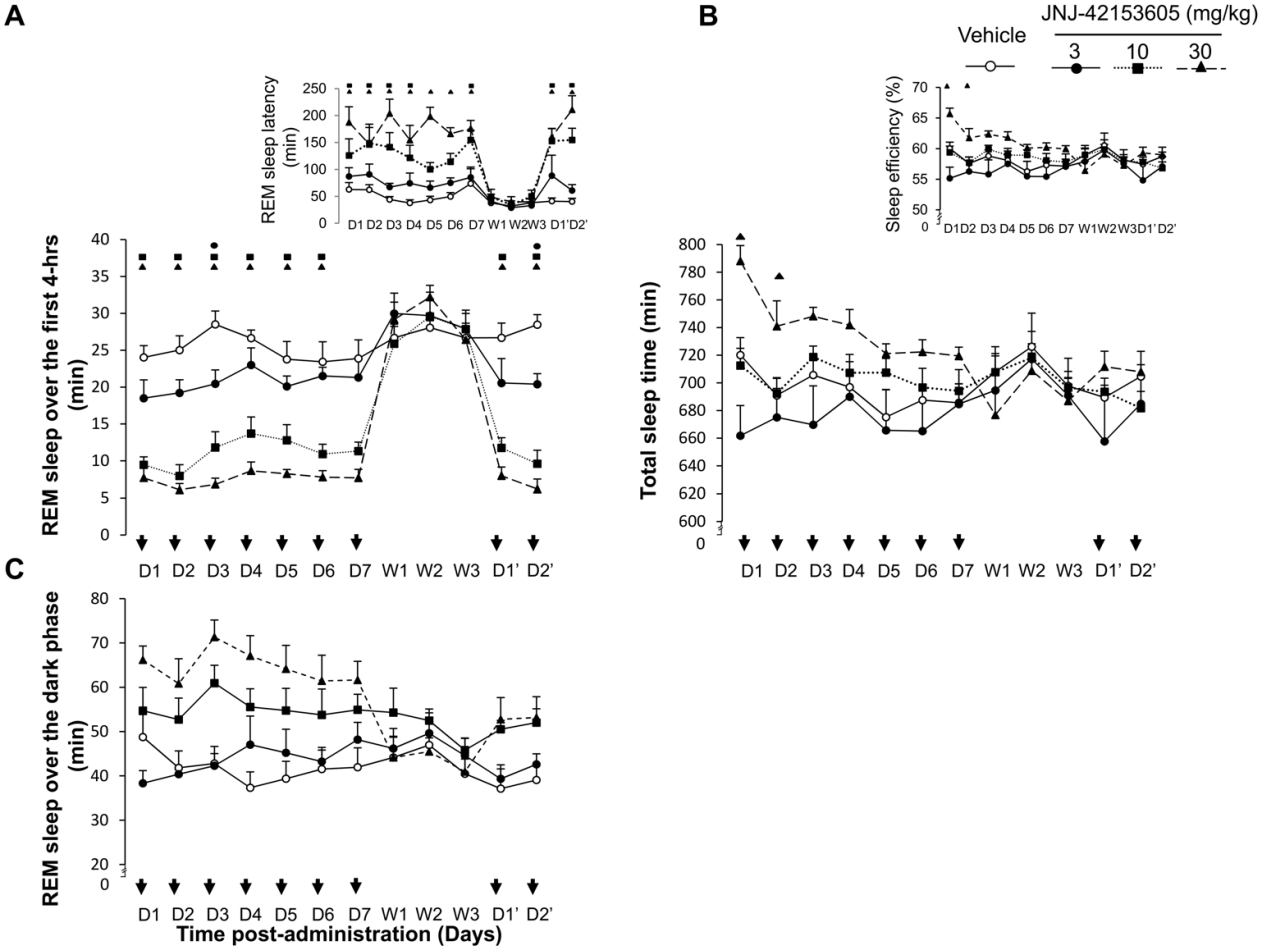
Sleep parameters following repeated dosing with JNJ-42153605 (3, 10, 30 mg/kg) or vehicle on experimental days (D1 first treatment day till D7 seventh treatment day), withdrawal days (W1 first withdrawal day till W3 third withdrawal day) and re-administration days after withdrawal (D’1 and D’2). Total sleep time expressed in minutes (Panel A) and sleep efficiency expressed in percentage (Panel A upper inset). REM sleep amount during the first 4-h of recording session after repeated administration (Panel B) and REM sleep latency (Panel B upper inset). Total amount of REM sleep expressed in minutes that was recovered during the subsequent 10-h dark period (Panel C). The data are presented as means ± S.E.M. Significant differences between the vehicle and JNJ-42153605 group are indicated by the drug-dose symbol.

JNJ-42153605 consistently decreased REM sleep and intermediate state during the first 4-h post-administration (“treatment x time” interaction: F(9, 212) = 3.6, p = 0.0003) and (“treatment x time” interaction: F(9, 212) = 5.9, p < 0.0001), respectively (Fig 6A). Concomitant to changes in REM sleep, JNJ-42153605 consistently lengthened REM sleep onset latency (F(3, 28) = 11.8, p < 0.0001) (Fig 6A, inset). Interestingly, JNJ-4215605 enhanced total sleeping time during the first 2 days following the administration (F(3, 28) = 5.4, p = 0.005) and (F(3, 28) = 2.9, p = 0.04) and increased sleep efficiency (Fig 6B and B inset) likely associated with the effects of the compound on deep sleep. JNJ-42153605 did not modify sleep parameters and total number of transitions from any sleep stage towards wakefulness.

Treatment days 2 till 7: Subsequent oral administration of JNJ-42153605 was accompanied with consistent reduction of REM sleep (“treatment x time” interaction: F(9, 212) = 3.07, p = 0.001) and enhancement of deep sleep (“treatment x time” interaction: F(9, 212) = 11.2, p = 0.0001). Repeated oral administration up to 7 days of JNJ-42153605 consistently reduced REM sleep and enhanced deep sleep during the first 4-h post-administration (Fig 6A). JNJ-42153605 maintained efficacy on REM sleep onset latencies over repeated treatment (Fig 6A, inset). Interestingly, JNJ-42153605 enhanced sleep maintenance and sleep efficiency, particularly with the higher dose of the compound (Fig 6B, inset), which may suggest a potential for improving sleep quality at early stage of the treatment.

Withdrawal days 8 till 10: During discontinuation of JNJ-42153605, the amount of different vigilance states was normalized and sleep parameters returned to baseline at cessation of treatment indicating that discontinuation of the compound did not result in withdrawal phenomena and sleep disturbances (Fig 6A and 6B).

Treatment days 11 till 12: In the subsequent re-administration 2 days, JNJ-42153605 elicited consistent enduring modifications of sleep-wake architecture similar to those observed in the initial subchronic treatment (Fig 6A and 6B). JNJ-42153605 reduced REM sleep during the first 4-h post-treatment (“treatment x time” interaction: F(9, 212) = 3.22, p = 0.001) and (“treatment x time” interaction: F(9, 212) = 3.26, p = 0.001), respectively, as well as lengthened REM sleep onset latency (F(3, 28) = 5.66, p = 0.003) and (F(3, 28) = 19.8, p < 0.0001).

During the 10-h dark period, total REM sleep amounts in rats treated with JNJ-42153605 showed significantly higher REM sleep recovered than the control vehicle treated animals (Fig 6C). Overall, the repeated recovery effects that were seen predominantly during the dark (active) phase did not persist in the subsequent light phase.

## Discussion

In the present study, JNJ-42153605 showed high affinity, potency and selectivity at mGluR2. We further report perspective on the allosteric binding site of mGluR2, the molecular docking analysis and the specificity of JNJ-42153605 at the mGluR2 allosteric binding site as compared with other mGluRs.

### Proposed binding mode of JNJ-42153605 at the mGluR2

Multiple mutagenesis studies concluded the presence of the 7TM allosteric binding site at mGluR2 [38–42]. In a recent report from our labs we published results from a large site directed mutagenesis study to clarify the binding mode of multiple mGluR2 PAMs [38]. JNJ-42153605 was part of that study although results were not included in the manuscript for reasons of space. That work confirmed a role for mutants F643A, H723V, L732A, N735D, W773A, and F776A on the PAM activity of JNJ-42153605. Recently, the mGluR1 and mGluR5 crystal structures have surpassed mutagenesis data by revealing the specifics of ligand receptor interaction. Comparing the binding mode of JNJ-42153605 (see S2 Fig) reveals good shape and volume overlap with that of FITM in the mGluR1 structure. However, in the case of the mGluR5, Mavoglurant penetrates deeper into the receptor due to a single amino acid difference (P655^3.36a.40c^ in mGluR5). This amino acid opens a narrow channel suitable for binding of the aryl acetylene motif which is common in mGlu5 allosteric modulators. The same position in mGlu2 corresponds to F643 which does not allow the ligand to bind so deep into the receptor. Hence, small changes in amino acids can make a difference to the binding site and contribute to specificity.

Comparison of the binding site amino acids (see S3 Fig) can further explain the specificity of JNJ-42153605. In particular, a key hydrogen bond is formed between the ligand which acts as acceptor and N735^5.47a.47c^ which behaves as donor. Interestingly, this amino acid is an aspartate in mGlu3 and hence being deprotonated is unable to act as an H-bond donor, or may have a different adjacent water network. Considering the structure activity relationships of mGluR2 PAMs and the presence of an H-bond acceptor in this position, along with the small adjacent substituent (in this case CF_3_) is crucial for activity. Hence it seems likely that this key interaction with N735^5.47a.47c^ and the insertion of the small hydrophobic substituent towards S731^5.42a.43c^, is likely a key interaction motif of mGlu2 PAMs. The combination of shape complementarity, changes in pocket volume, and specific amino acid interactions contribute to the specificity of JNJ-42153605.

Recent work in our labs has focused on the detailed characterization of the radioligand [^3^H]-JNJ- 46281222 [43]. A detailed modelling study was included in that work which took advantage of SAR knowledge and experimental mutagenesis data reported therein and previously [38], to derive a binding mode for the JNJ-46281222. In this report we show that JNJ-42153605 competitively displaces [^3^H]-JNJ-46281222 in occupancy assays. This is understandable given the similarity in structure between the two ligands, and the similar predicted binding modes (see S4 Fig). The ligands overlap very well and form the same interactions between the cyclopropylmethyl groups, the scaffold, the H-bond to N735^5.47a.47c^ as well as the CF_3_ group interaction towards S731^5.42a.43c^ in TM5. There is small structural deviation in the ligand position at the extracellular side of the binding site. This is as expected due to the larger cavity and the increased flexibility of JNJ-46281222 compared to JNJ-42153605 which arises due to the methylene spacer between the scaffold and the piperidine nitrogen.

Thus, this docking modeling was highly valuable for the sleep-wake study: by better defining the JNJ-42153605 compound in its role as an appropriate mGluR2 PAM, the in vivo outcomes and comparison with the LY354740 agonist is more reliable.

The present in vivo findings indicate that acute activation of mGluR2 by the agonist LY354740 and acute allosteric modulation by the specific PAM JNJ-42153605 consistently and dose-dependently reduced REM sleep and intermediate stage during the first 4-h post-administration and lengthened REM onset latency.

### Activation of mGluR2 affects REM sleep state

The glutamatergic system is fundamental for physiological brain processes and any dysfunction in glutamate neurotransmission may play an important role in the pathophysiology of psychiatric disorders. Glutamatergic tone exhibits a circadian burst across sleep wake states, being maximal in the orbitofrontal cortex and rostromedulla nuclei during wakefulness and REM sleep [18,44]. Therefore, alteration of glutamatergic neurotransmission is expected to affect the organization of vigilance states. Accordingly, local administration of glutamate in the brainstem pontine nuclei elicited REM sleep [45]; while blockade of NMDA increased the homeostatic need for sleep through neuroendocrine pulses controlled by hypothalamic sleep centres [46]. mGluR2 is of particular interest because of its unique distribution and regulatory roles in synaptic neurotransmission [25,26,47]. The effects of mGluR2 agents on sleep has been examined by several research groups, and a great consistency across all these studies has been found with respect to the suppressing effect on REM sleep. Systemic or central activation of mGluR2 selectively suppress REM sleep and prolong its onset latency [20,21,23], whereas blockade of mGluR2 promoted wakefulness [21,48]. The specificity of the changes has been supported by the findings in mGluR2 (-/-), in which activation of mGluR2 specifically suppressed REM sleep in WT animals but not mGluR2 (-/-) mouse strain [20]. Thus, our present results are consistent with the profile of central activation of mGluR2, resulting in a common changes in sleep-wake organization of mice and rats i.e. dose-response inhibition of REM sleep occurrence and lengthening of REM sleep onset latency.

There have been attempts to further dissect the neural basis of the effects of mGluR2 agents on sleep. On one hand, acetylcholine, serotonin and GABA neurotransmission play a pivotal role in the regulation of sleep behaviour, and mGluR2 activation modulates the levels of these neurotransmitters in several brain regions [10,49,50]. Consequently, activation or inhibition of the GABAA receptors decreased and increased REM sleep, respectively [28].

On the other hand, mGluR2 are highly expressed in the basolateral and central nuclei of the amygdala [25–27,51]. These limbic structures send direct projections to brain stem nuclei known to control the mechanisms of sleep and arousal [28,52,53]. Activation of mGluR2 within the basolateral amygdaloid region selectively suppressed REM sleep [23]. Therefore, activation of mGluR2 might affect the organization of vigilance states through other pathways than direct glutamatergic system. A promising candidate is the sublaterodorsal nucleus (SLD), which contains glutamatergic neurons that are crucial for the generation of atonia during REM sleep [54]. Acetylcholine is thought to participate in the activation of these descending atonia pathways as increases in glutamatergic input to SLD neurons occur during REM sleep when the levels of acetylcholine in the dorsal pons are highest [55]. LY354740 has been shown to inhibit the electrically-evoked endogenous acetylcholine release in brain slices [56] as well as the NMDA-evoked [3H]-choline release from synaptosomes and acetylcholine release in brain slices [57]. While depletion of acetylcholine inhibits REM sleep and blocking acetylcholine degradation promotes REM sleep [58], it is possible that reduced REM sleep may result from LY354740-induced inhibition of acetylcholine release.

### mGluR2 agonist but not PAM elicited tolerance to its primary functional activity on REM sleep

During repeated dosing, the efficacy of LY354740 rapidly vanished as from day 3 onwards indicating rapid development of tolerance to REM sleep suppression. In contrast, the specific mGluR2 PAM elicited enduring significant inhibitory effects on REM sleep measures up to day 7. During discontinuation of JNJ-42153605, all vigilance states were normalized indicating a lack of withdrawal adverse side effects at cessation of the treatment. In the subsequent re-administration days, JNJ-42153605 consistently suppressed REM sleep variables indicating that no rapid development of tolerance to REM sleep suppression action took place.

There is evidence that agonists rapidly lose their efficacy after repeated dosing. Receptor desensitization and internalization are two early events that are thought to mark the onset of tolerance [59].

Our findings are consistent with the hypothesis that mGluR2 agonists function best as acute, time-limited interventions and highlighted a possible role of receptor desensitization and/or down regulation as a limiting factor for mGluR2/3 agonist therapies. However, caution should be taken as the expression levels, receptor activation, desensitization, internalization, recovery from desensitization of mGluR2/3 receptors were not assessed in the present study. Thus, the results obtained in this preclinical translational model shed lights on possible tolerance development to a primary functional activation with the mGluR2 agonist.

### REM sleep recovery was differently affected by LY354740 and JNJ-42153605

During the subsequent 10-h dark period, the increase gain in REM sleep over vehicle was greater in animals treated with JNJ-42153605 but not with LY354740.

Sleep is regulated by a homeostatic drive that increases sleep propensity in proportion to the duration of prior waking and a circadian drive that alternately promotes sleep and waking across the day [60]. Sleep loss is known to induce a compensatory increase that is proportional to the duration of sleep suppression and to the stage of sleep that was lost e.g. REM sleep [61]. LY354740 powerfully and specifically suppresses spontaneous REM sleep and its homeostasis recovery during the light and dark phases, respectively. The mechanisms underlying the reduced recovery of REM sleep after the administration LY354740 are not well understood. The basal forebrain is one key brain region implicated in sleep recovery after sleep loss [62,63], and it is possible that mGluR2/3 activation may act on the basal forebrain and its projections to the cortex to alter homeostatic need of REM sleep generation. Another potential mechanism that could explain the reduced ability of LY354740 to inhibit and to recover REM sleep in subsequent days could be related to diminished efficacy of mGluR2s trafficking including their internalization and desensitization in response to repeated stimulation as described earlier for the serotonin 5-HT7 receptor [64]. Future studies need to investigate the detailed molecular mechanisms and relationship between intrinsic agonist efficacy and agonist-induced internalization and/or desensitization, and to address in future clinical work whether the loss of efficacy after mGluR2 agonism may be related to clinical tolerance issues. Until such studies are performed, the current findings underscore possible unresponsiveness to long term use of mGluR2 agonists.

It is noteworthy that the JNJ-42153605-induced inhibition of REM sleep during the light phase was followed by a delayed compensatory increase during the following dark phase. This time course is consistent with molecular dynamics that can mediate genomic expression of proteins involved in sleep-wake regulatory nuclei including brainstem neurons, basal forebrain, thalamus and hypothalamus/preoptic area, that are thought to promote REM sleep. Consistent with these possibilities, mGluR2 activation modulates c-Fos expression markers of neuronal activation in sleep and wake regulatory areas such as the amygdala and thalamic nuclei [23,64–66]. Consistent with a homeostatically driven increase in REM sleep, the acute homeostatic increase in REM sleep response was preserved throughout the subchronic treatment and following withdrawal.

The ability of LY354740 to attenuate the homeostatic need for REM sleep, while JNJ-42153605 promoted recovery sleep raises the potential benefit from recovery sleep in relation to performance and cognitive function, which may be an important factor in differentiating these two compounds and related mechanism of action. These data do not preclude the possibility that other mGluRs including mGluR3 may also be implicated in the lack of REM sleep recovery. Nevertheless, the present data point clearly point to a differential timing of REM recovery to preserve the normal light-dark REM sleep distribution and suggest the mGluR2 signalling as a mechanism for REM sleep homeostasis.

### mGluR2 PAM had an early deep sleep promoting effect

Interestingly, JNJ-42153605 appears to enhance deep sleep, at least during the first days of treatment. JNJ-42153605 had positive effects on sleep continuity and efficiency, which may be beneficial at early stages of the treatment in psychiatric disorders associated with impaired sleep quality.

Sleep disturbances are prevalent in psychiatric diseases and poor sleep quality is associated with decrements in cognitive performance [67]. The anatomical and functional interactions between mGluR2 and 5HT2A receptors have been reported to form heterodimeric complex that modulate G-protein-mediated intracellular signaling differentially compared to mGluR2 and 5-HT-2A homomers [68–71], and both human and animal pharmacological studies demonstrated that an interaction with 5-HT2A receptor can improve comorbid sleep disturbance associated with mood disorders and schizophrenia [72–75]. Similarly, the functional interactions between mGluR2 and NMDA receptors signaling has been evidenced by the inhibitory effects of the more potent mGluR2 agonist LY379268 and the PAM CBiPES, which modulate the ketamine evoked increases in glutamate release in the medial prefrontal cortex [75], as well as histamine release the limbic brain regions [76] point towards a reduction in central excitatory levels.

Therefore, the early effects of JNJ-42153605 on deep sleep and sleep efficiency suggest a putative functional interaction response that may result in a greater early benefit on sleep regulating emotional brain reactivity and functioning.

### Effectiveness of different mGluR2 PAMs on sleep-wake pharmacodynamics index

The Table 1 shows a summary of half-lives (T_1/2_), brain receptor occupancy and the relative contribution of mGluR2 versus 5-HT2A receptor in the sleep-wake pharmacodynamics model of published mGluR2 PAMs (JNJ-40411813, JNJ-40068782 and JNJ-42153605). All mGluR2 PAMs showed comparable mean T_1/2_ values (ranging from 2.3 to 2.4-h) following oral administration in rats.

**Table 1:** Summary of halve-lives (T_1/2_ expressed in h), brain receptor occupancy (Efficacy Dose “ED_50_” expressed in mg/kg) and pharmacodynamics effect of published mGluR2 PAMs (JNJ-40411813, JNJ-40068782, JNJ-42153605) on sleep measures in rats (Lowest Active Dose “LAD” expressed in mg/kg) [30,31,77].

	JNJ-40411813	JNJ-4139782	JNJ-42153605
**T1/2 (h)**	2.3 ± 0.5	2.4 ± 0.3	2.3 ± 0.5
**Occupancy ED50 (mg/kg)**			
mGluR2	16	5.5	5.5
5HT2A	9.5	1.6	> 40
**Pharmacodynamic index ED50 (mg/kg)**			
Deep sleep	3	3	3
REM sleep	10	10	10

#### JNJ-40411813

JNJ-40411813 is an mGluR2 PAM with a moderate antagonist activity at the 5HT2A receptor, dose-dependently decreased REM sleep and increased deep sleep in rats [77]. Over a period of 4-h, JNJ-40411813 was effective at the Lowest Active Dose (LAD) of 3 mg/kg for REM sleep and 10 mg/kg for deep sleep. This corresponds to plasma levels of 50–180 ng/ml and 25–45% mGluR2 occupancy. Exposure levels of JNJ-40411813, that were needed to maintain effects on REM sleep at the lowest efficacious plasma level in this model was 40 ng/ml, corresponding to 50% receptor occupancy.

#### JNJ-41329782 versus JNJ-40411813

JNJ-41329782 acts as a mixed mGluR2 PAM-5-HT2A antagonist with similar affinity and activity at both receptors [31]. JNJ-41329782 is brain penetrant and occupies rat brain mGluR2 with an ED_50_ of 5.5 mg/kg, corresponding to a plasma concentration of 850 ng/ml, while the 5-HT2A occupancy was reached at lower plasma concentrations (EC_50_ 54 ng/ml). JNJ-41329782 is comparable in potency to JNJ-40411813, however, a more than 3-fold difference in corresponding ED_50_ was found for 5-HT2A receptor occupancy after oral administration (1.6 and 9.5 mg/kg, respectively). JNJ-41329782 seems somewhat more potent than JNJ-40411813, although both compounds are very comparable in their pharmacological profile. JNJ-41329782 had no activity towards other mGluR subtypes up to a concentration of 10 μM. Similar to JNJ-40411813, JNJ-41329782 reduced REM sleep during the first 4-h post-administration with a LAD of 3 mg/kg; corresponding to ~30% receptor occupancy and enhanced deep sleep with a LAD of 10 mg/kg, corresponding to plasma levels of 320 ng/ml.

#### JNJ-42153605 versus JNJ-40411813

Compared to JNJ-40411813, JNJ-42153605 is about 10-fold more potent in vitro on mGluR2. JNJ-42153605 showed a binding as well as functional mGluR2 affinity in the range of 15 nM. JNJ-42153605 increased glutamate efficacy up to 3-fold and enhances glutamate potency up to 25-fold at both the cloned human and rat mGluR2. In contrast to JNJ-40411813, which shows moderate 5-HT2A antagonist activity, JNJ-42153605 shows >100-fold selectivity versus other targets. Similar to JNJ-40411813, JNJ-42153605 suppressed REM sleep over a period of 4-h at a LAD of 3 mg/kg and enhanced deep sleep at 10 mg/kg, which corresponds to plasma levels of 40–170 ng/ml and 50–90% mGluR2 occupancy.

Overall, a comparative analysis of the different, published mGluR2 PAMs JNJ-40411813, JNJ-40068782, JNJ-42153605 shows a functional effectiveness on sleep wake pharmacodynamics at similar LAD.

### Functional significance

There has been pharmacological evidence indicating the potential of mGluR2 agents in animal models of anxiety and stress [5,78]. Sleep is regulated by multiple ascending and descending pathways including the limbic structure, which is critically implicated in the control of anxiety and emotion [79]. Sleep disturbance are prevalent symptoms in depression, anxiety and stress related disorders and improvement of sleep has often been considered as one of the first signs of impending recovery [80–83]. Additionally, sleep-EEG variables such as elevated REM density and REM latency are used as fundamental biomarkers and diagnostic criteria in major depressive disorders [84,85]. Moreover, a sustained suppression of REM sleep variables and lengthening of REM latency are common features found in healthy, depressed subjects and laboratory animals after chronic treatment with clinically effective antidepressants [86–92]. Therefore, the intimate relationship between glutamate signalling and sleep mechanisms highlights the value of sleep-wake measurements as a reliable sensitive index of the mGluR2 target engagement. Potentiating the mGluR2 response to glutamate by using selective PAMs may offer advantages over orthosteric mGluR2 agonists to achieve superior mGluR2 selectivity [5,93], which may translate to an improved efficacy and tolerability profile in the clinic. Additionally, potentiators may exhibit lower potential for the induction of receptor desensitization and tolerance that can occur with GPCR agonists during long-term dosing. The present results suggest that mGluR2 modulators may have superior therapeutic efficacy over agonists to counter-act excessive glutamate flow in psychiatric disorders associated with REM sleep overdrive.

Overall, repeated treatment with LY354740 elicited tolerance phenomena to its primary acute functional effect, whereas JNJ-42153605 resulted in sustained efficacy on sleep measures in rats. JNJ-42153605 promoted homeostatic recovery sleep and had an early beneficial effect on sleep efficiency, which may serve as an early add-on treatment of impaired sleep quality in psychiatric disorders commonly associated with REM sleep overdrive. From the translational perspective, the present findings should raise awareness that long term therapeutic application of an mGluR2 agonist, but not of a PAM might lead to tolerance development to its primary acute functional activity.

## Supporting Information

S1 Fig Sequence Alignment used to construct mGluR2 7TM model. (TIF)

S2 Fig Comparison of the predicted JNJ-42153605 binding mode with that of NAMs in the mGluR1 (top, yellow) and mGluR5 (bottom, orange) X-ray structures. **Top**: mGluR1 (yellow) compared to mGluR2 model (purple). Position of allosteric ligand and selected amino acid side chains can be seen. mGluR1 X-ray structure is from PDB code 4OR2. **Bottom**: mGluR5 (orange) compared to mGluR2 model (purple). Position of allosteric ligand and selected amino acid side chains can be seen. mGluR5 X-ray structure is from PDB code 4OO9.
(TIF)

S3 Fig Binding site amino acid comparison, human mGluR2 is highlighted in the red box. Amino acids are non-sequential and identified by selecting within 6Å radius of ligand in mGluR1 and mGluR5 crystal structures, selected examples from mGluR2 are labelled.
(TIF)

S4 Fig Overlap of the proposed binding mode of JNJ-42153605 (magenta) binding mode with that of JNJ-46281222 (blue). (TIF)
