# Supplementary figures and images for: mGlu2 Receptor Agonism, but Not Positive Allosteric Modulation, Elicits Rapid Tolerance towards Their Primary Efficacy on Sleep Measures in Rats
Source: PLoS One. 2015 Dec 11;10(12):e0144017. doi: 10.1371/journal.pone.0144017 (PMC4684355; doi:10.1371/journal.pone.0144017)

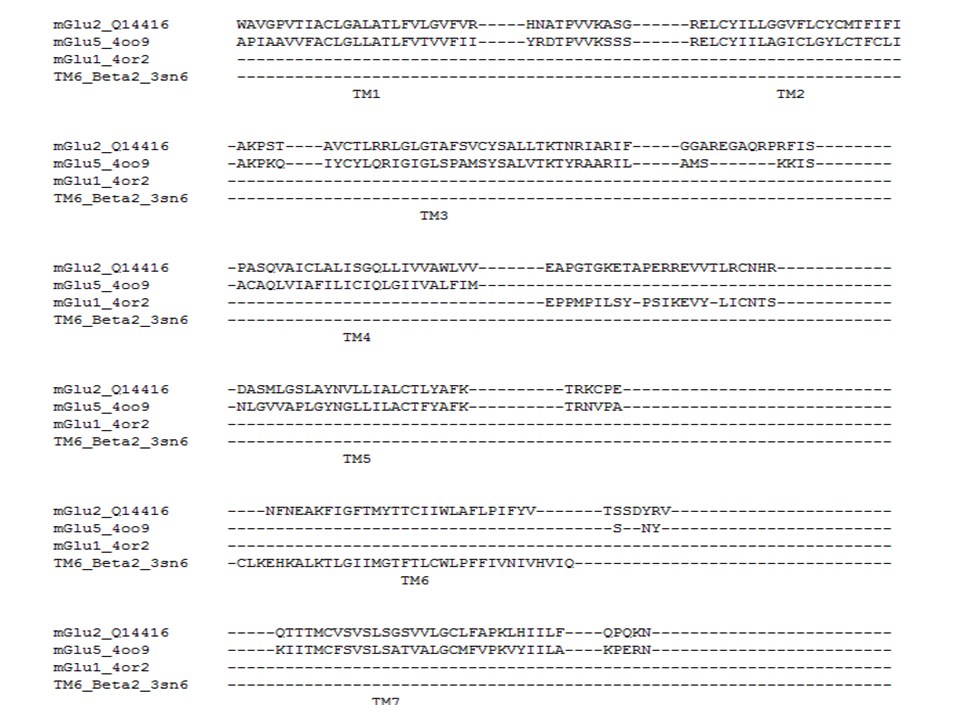

Supplement: S1 Fig — (TIF) [file pone.0144017.s001.tif]

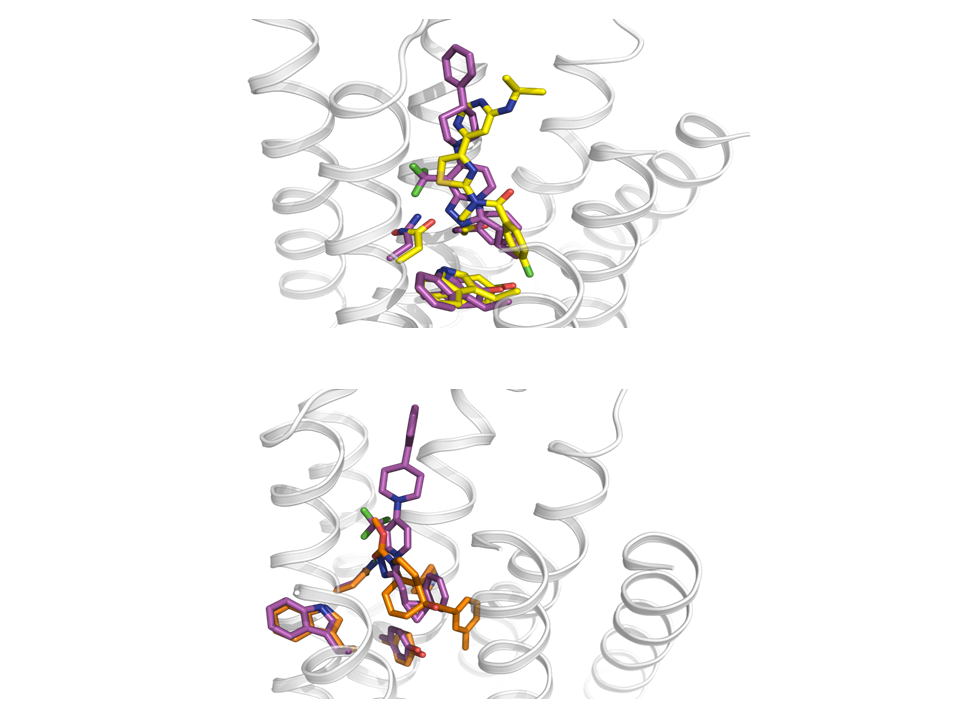

Supplement: S2 Fig — Top: mGluR1 (yellow) compared to mGluR2 model (purple). Position of allosteric ligand and selected amino acid side chains can be seen. mGluR1 X-ray structure is from PDB code 4OR2. Bottom: mGluR5 (orange) compared to mGluR2 model (purple). Position of allosteric ligand and selected amino acid side chains can be seen. mGluR5 X-ray structure is from PDB code 4OO9. (TIF) [file pone.0144017.s002.tif]

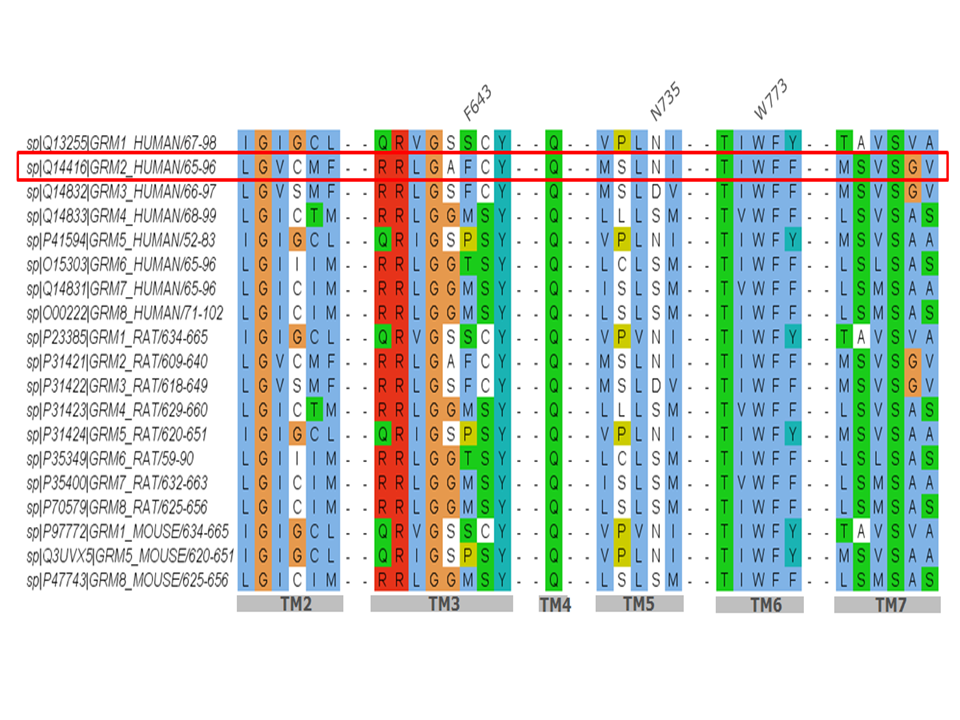

Supplement: S3 Fig — Amino acids are non-sequential and identified by selecting within 6Å radius of ligand in mGluR1 and mGluR5 crystal structures, selected examples from mGluR2 are labelled. (TIF) [file pone.0144017.s003.tif]

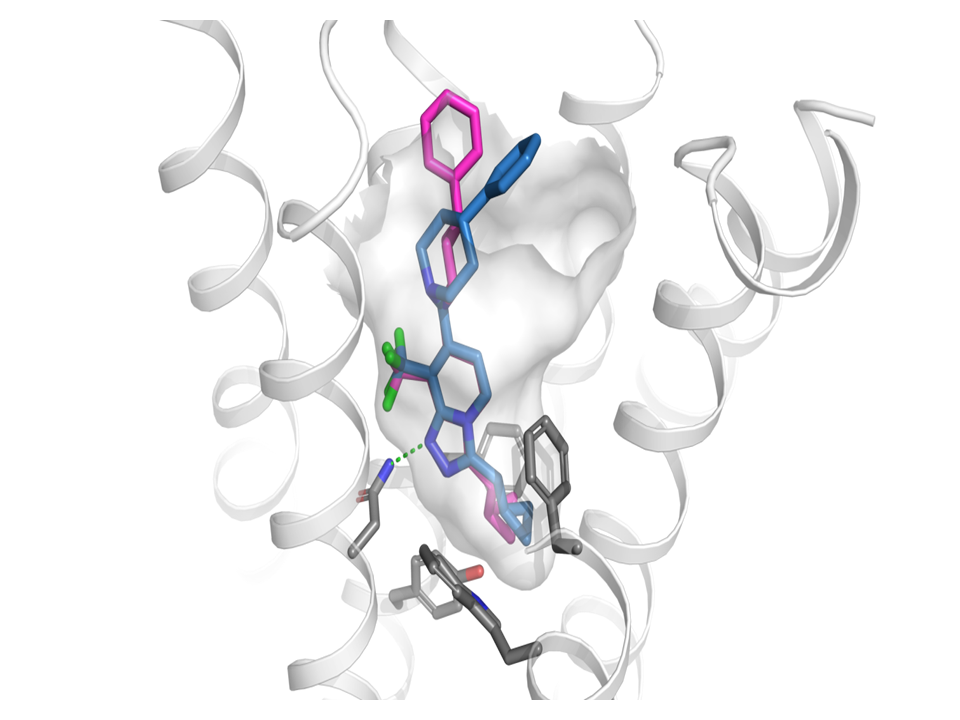

Supplement: S4 Fig — (TIF) [file pone.0144017.s004.tif]
